# Assessing causal relationships between gut microbiota and asthma: evidence from two sample Mendelian randomization analysis

**DOI:** 10.3389/fimmu.2023.1148684

**Published:** 2023-07-19

**Authors:** Rong Li, Qi Guo, Jian Zhao, Wenhui Kang, Ruoyu Lu, Zichong Long, Lili Huang, Yiting Chen, Anda Zhao, Jinhong Wu, Yong Yin, Shenghui Li

**Affiliations:** ^1^ Ministry of Education-Shanghai Key Laboratory of Children’s Environmental Health, School of Public Health, Shanghai Jiao Tong University School of Medicine, Shanghai, China; ^2^ School Health Department, Shanghai Center for Disease Control and Prevention, Shanghai, China; ^3^ Shanghai Ninth People’s Hospital, Shanghai Jiao Tong University School of Medicine, Shanghai, China; ^4^ Shanghai Children’s Medical Center, Shanghai Jiao Tong University School of Medicine, Shanghai, China

**Keywords:** Mendelian randomization, gut microbiota, asthma, childhood-onset asthma, causality

## Abstract

**Background:**

Accumulating evidence has suggested that gut microbiota dysbiosis is commonly observed in asthmatics. However, it remains unclear whether dysbiosis is a cause or consequence of asthma. We aimed to examine the genetic causal relationships of gut microbiota with asthma and its three phenotypes, including adult-onset asthma, childhood-onset asthma, and moderate-severe asthma.

**Methods:**

To elucidate the causality of gut microbiota with asthma, we applied two sample Mendelian randomization (MR) based on the largest publicly available genome-wide association study (GWAS) summary statistics. Inverse variance weighting meta-analysis (IVW) was used to obtain the main estimates; and Weighted median, MR-Egger, Robust Adjusted Profile Score (MR-RAPS), Maximum likelihood method (ML), and MR pleiotropy residual sum and outlier (MR-PRESSO) methods were applied in sensitivity analyses. Finally, a reverse MR analysis was performed to evaluate the possibility of reverse causation.

**Results:**

In the absence of heterogeneity and horizontal pleiotropy, the IVW method revealed that genetically predicted *Barnesiella* and *RuminococcaceaeUCG014* were positively correlated with the risk of asthma, while the association between genetically predicted *CandidatusSoleaferrea* and asthma was negative. And for the three phenotypes of asthma, genetically predicted *Akkermansia* reduced the risk of adult-onset asthma, *Collinsella* and *RuminococcaceaeUCG014* increased the risk of childhood-onset asthma, and *FamilyXIIIAD3011group*, *Eisenbergiella*, and *Ruminiclostridium6* were correlated with the risk of moderate-severe asthma (all *P*<0.05). The reverse MR analysis didn’t find evidence supporting the reverse causality from asthma and its three phenotypes to the gut microbiota genus.

**Conclusion:**

This study suggested that microbial genera were causally associated with asthma as well as its three phenotypes. The findings deepened our understanding of the role of gut microbiota in the pathology of asthma, which emphasizes the potential of opening up a new vista for the prevention and diagnosis of asthma.

## Background

The human gastrointestinal system is a habitat for trillions of bacteria and archaea that live in mutual symbiosis with an individual’s body, and this symbiotic relationship is crucial to human health (1). Gut microbes are involved in a variety of physiological functions, including the maintenance of metabolic stability, the regulation of immune response, the resistance to infection, and et al (2). The gut microbiota has been confirmed to be implicated in disease susceptibility and progression by an increasing number of studies, and it is now regarded as an endocrine organ (3). Numerous connections between the gut microbiota and other complex features have been discovered with the advancement of high-throughput sequencing technologies and platforms (4).

As one of the most common chronic respiratory diseases, asthma affects around 334 million people worldwide, covering all age groups and usually originating from childhood (5). To date, the pathogenesis of asthma has not been fully elucidated yet, and the contribution of genetic factors was estimated to be ranged from 25% to 80% (6). Recent genome-wide association studies (GWAS) identified 18 asthma-associated genomic loci including five new loci at 5q31.3, 6p22.1, 6q15, 12q13.3, and 17q21.33 (7). And the 22 distinct genome-wide-significant single nucleotide polymorphisms (SNPs) corresponding to 18 loci can explain 3.5% of the variance in asthma liability (7). As regards the phenotypes of asthma, existing evidence revealed that childhood-onset asthma was highly associated with at least two independent loci, 17q12-21 and filaggrin (FLG) locus; and a relative moderate association was also found between adult-onset asthma, moderate-severe asthma and genes, which are largely a subset of those associated with childhood-onset asthma (8).

A growing body of literature has revealed that asthma comprises a range of heterogeneous subtypes with significant variance in clinical characteristics and course, and the heterogeneity should be based, at least partly, on different genetic origins with respect to varied asthma subtypes (9). Therefore, examing subtype-specific genetics, along with identifying the shared genetic factors underlying asthma and its coexistent diseases, should be of importance in clarifying the heterogeneity of asthma. By using the UK Biobank and 23andMe data, two recent studies obtained largely consistent findings that there was not only genetic overlap (eg, IL1RL1, HLA-DQA1) between childhood asthma and adult asthma but also subtype-specific components, providing evidence that the heterogeneity of asthma is related to distinct genetics (10, 11). These research advances bring fresh perspectives on the genetic genesis of asthma.

There is a growing interest in the relationship between gut microbiota and asthma (12, 13). The “hygiene hypothesis” is the initial theory to propose a connection between microbiota and allergy (12), and then followed by the concept of the “gut-lung axis”, which states that a dysbiosis of the gut microbiota might cause airway disease by changing the immune response (13). Asthma is mainly mediated by type I hypersensitivity reactions, accompanied by T helper (Th) subgroup and Th1/Th2 immune imbalance (5). Several studies have confirmed the role of the microbiota in regulating T-cell homeostasis (14–16). Data suggested that *Bacteroides fragilis* can modulate Th1/Th2 balance, *Segmented filamentous bacteria* can directly stimulate Th17 cell differentiation, and *Clostridium* is involved in the induction of Treg production (14–16). Tregs are a subpopulation of T cells that play key roles in modulating immune system activity and in maintaining tolerance to self-antigens (17). A previous study among preschool children diagnosed with asthma provided the evidence of gut microbiota dysbiosis that the reduction of *Lachnospira* was potentially linked to asthma (18). In particular, the opposite changes in the relative abundance of *Lachnospira* and *Clostridium in the first three months after birth* at three months of life play a role in promoting the development of a childhood-onset asthma phenotype (18). Moreover, in clinical studies, the connections between microbial dysbiosis and asthma characteristics have involved adult asthmatics, who have a higher concentration of histamine-secreting bacteria in their gut than healthy subjects, indicating these gut microbes may affect manifestations of allergic asthma (19).

Nevertheless, above findings from observational studies make it difficult to infer true causality, given the presence of reverse causality and the potential confounding factors. With the rapid increase in genetic data on microbiota and complex diseases, Mendelian randomization (MR) has gained widespread use in recent years. MR has a unique advantage in exploring the potential causal relationship between two traits based on mendelian laws of inheritance (20). In this study, we conducted a two sample MR study based on recently published large GWAS summary datasets to investigate the genetic causal link between gut microbiota, asthma and its phenotypes, and to identify particular groupings of pathogenic bacteria (4, 21, 22).

## Methods

This MR study was undertaken following a framework as delineated in **Figure S1**. The approach is conducted based on three assumptions: 1) genetic variation used as instrumental variable (IV) is associated with exposure; 2) genetic variation is independent of confounding factors, and 3) genetic variation affects outcome risk only through the exposure of interest and not through other pathways (20).

## Data sources

### Gut microbiota

MiBioGen is an international consortium dedicating the better understanding to genetic architecture of gut microbiota. It has collected information from 24 population-based cohorts with a total of 18,340 individuals. Each cohort investigated the gut microbiota *via* 16S rRNA sequencing and participants were genotyped by using full-genome SNP arrays (4). The HRC 1.0 or 1.1 reference panel was employed for genotyping imputation. Then, covariates such as age, sex, technical covariates, and genetic principal components were taken into account, and association analysis was performed by using Spearman correlation (4). The gut microbiota GWAS summary data includes a total of 131 genera; and 119 genera were included in our study as exposures, with the exclusion of 12 unknown genera. Detailed information on the classification of gut microbiota can be seen in **Additional file 1: Table S1**.

### Asthma, adult-onset asthma and childhood-onset asthma

GWAS summary datasets for asthma, adult-onset asthma and childhood-onset asthma were obtained from the largest sample of recent publications, and the source of cases and controls was the UK Biobank (22). Asthma cases in the UK Biobank were determined by subjects’ responses to the question “whether they had been diagnosed with asthma by a doctor” (22). In the asthma GWAS data, a total of 394,283 people of European ancestry were included, comprising 46,802 asthmatics and 347,481 controls, childhood-onset asthma was defined as onset age of asthma ≤12 years (including 9,676 cases and 347,481 controls), whereas adult-onset asthma was defined as onset age ≥26 years (including 22,296 cases and 347,481 controls) (22).

### Moderate-severe asthma

As for moderate to severe asthma, a recent large-scale European ancestry GWAS summary dataset was used, which includes 5,135 moderate-to-severe asthma cases and 25,675 controls (21). The consortia for cases and control sources were the Genetics of Asthma Severity and Phenotype study (GASP), the Unbiased Biomarker Prediction of respiratory diseases outcomes project (U-BIOPRED), and the UK Biobank. Clinical records were used to evaluate patients in GASP and U-BIOPRED in accordance with British Thoracic Society criteria since 2014. Additionally, cases of moderate-severe asthma of moderate-severe asthma in UK Biobank are determined by a doctor’s diagnosis (21).

All datasets were taken from previously published literature, exempt from ethical approval. Details of the GWAS summary datasets for gut microbiota, asthma, and asthma phenotypes can be seen in **Additional file 1: Table S2**.

### Selection of genetic instrument

The following quality control procedures were used to choose the appropriate genetic IV in order to guarantee the validity and correctness of the conclusions about the causal link between the gut microbiota and asthma risk. First, with reference to most MR studies on gut microbiota (23, 24), we set the significance level at *P*<1.0×10^-5^ to identify enough candidate instruments due to the minimal number of loci found for gut microbiota. Second, to identify the independent SNPs assorted randomly during gestation, the clumping process (R^2^ <0.001, and clumping distance=10,000 kb) was conducted to assess the linkage disequilibrium (LD). Third, palindromic SNPs were removed since the GWAS of gut microbiota did not provide the effective allele frequency so we are unable to determine whether these SNPs were aligned in the same direction for exposure and outcome or not. Four, to reduce the heterogeneity and avoid the pleiotropy, MR Pleiotropy Residual Sum and Outlier (MR-PRESSO) methods were used to identify the horizontal pleiotropic outliers. Finally, the F-statistics (BETA^2^/SE^2^) for each IV of the gut microbiota was calculated as a measure of instrument strength (25). Generally, F-statistics > 10 were set as the threshold of strong IVs, otherwise, the IV is considered to have a weak association with exposure and would be therefore excluded. The detailed information on the IVs is displayed in **Additional file 2: Table S3**.

### Statistical analysis

In the main analysis, we obtained estimates from inverse variance weighting meta-analysis (IVW), which aggregate the Wald values for each SNP and derive the overall estimates of the effect by using meta-analysis (26). The random-effect IVW would be employed if there is heterogeneity among the SNPs included in each analysis (27).

### Sensitivity analyses

To evaluate the sensitivity of genetic causal effects, Weighted median, MR-Egger, Robust Adjusted Profile Score (MR-RAPS), Maximum likelihood method (ML), and MR-PRESSO methods were applied, which would promise to provide evidence of validity under different conditions (28–33). The PhenoScanner database (http://www.phenoscanner.medschl.cam.ac.uk/) was also searched to investigate whether the selected SNPs are associated with confounding traits (BMI, tobacco or alcohol exposure, and pulmonary function) at a significance level of 1 × 10^–5^, and we re-run the analysis after dropping these SNPs. Moreover, the Cochran Q statistic and I^2^ statistic were used to test the heterogeneity. To determine if a specific genetic locus have an impact on random estimates, the leave-one-out sensitivity method was used. Scatterplots, forest plots, and funnel plots were created to further demonstrate the sensitivity of the results.

### Power calculations

Power calculations for this MR were performed on the website: mRnd (http://cnsgenomics.com/shiny/mRnd) (34).

The threshold for statistical significance was set at 4×10^-4^ (*P*=0.05/119) using a Bonferroni-adjusted P-value. If 4×10^-4^<*P*<0.05, this was considered suggestive of evidence for a potential association. All the analyses and relevant figures were made by R 3.6.5, using the “TwoSampleMR (0.5.6)”, “MR-PRESSO (1.0)”, and “mr.raps (0.2)” packages. Reporting follows the STROBE-MR statement (35).

## Results

The characteristics of the selected SNPs for each gut microbiota are presented in **Additional file 2: Table S3**. Since the gut microbiota GWAS summary statistics didn’t report the effective allele frequency and variance explained (R^2^), the range of possible R^2^ values was shown, and we calculated the OR detectable with 80% power, detailed information can be seen in **Additional file 2: Table S4**.

### Effects of genetically predicted gut microbiota on asthma

Based on the IVW method, 3 genera of genetically predicted gut microbiota were found to be related to the risk of asthma (**Table 1**). The MR analysis showed that genetically predicted *Barnesiella* increased the risk of asthma (*OR*=1.097; 95% confidence interval [*CI*]=1.034–1.163; *P*=2.10E-03), the similar results were also obtained in the ML, MR Egger, RAPS and MR-PRESSO methods. Similarly, genetically predicted *RuminococcaceaeUCG014* increased the risk of asthma (*OR*=1.121; 95%*CI*=1.039–1.210; *P*=3.06E-03). And ML, RAPS and MR-PRESSO also yielded the same results. By contrast, genetically predicted *CandidatusSoleaferrea* was shown to be a protective factor for asthma (*OR*=0.934; 95%*CI*=0.884–0.987; *P*=1.58E-02), and both the ML and MR-PRESSO methods supported the finding.

**Table 1:** MR results of causal relationships between the Gut microbiota and Asthma, and its phenotypes risk.

*Gut microbiota*	*MR Results*						*Heterogeneity*			*Horizontal pleiotropy*				
	*Methods*	*SNPs*	*Beta*	*SE*	*P-* *value*	*OR (95% CI)*	*Cochran’s Q*	*I^2^*	*P-* *value*	*Egger* *intercept*	*SE*	*P-* *value*	*RSSobs*	*P-* *value*
**Asthma**														
**Barnesiella**	IVW	13	0.092	0.030	2.10E-03	1.097(1.034-1.163)	12.410	3.3	0.413	-0.012	0.008	0.160	14.429	0.448
	ML	13	0.096	0.031	1.71E-03	1.101(1.037-1.169)	11.998	0	0.446					
	MR Egger	13	0.234	0.098	3.69E-02	1.263(1.042-1.532)	10.144	0	0.518					
	Weighted median	13	0.078	0.043	7.00E-02	1.081(0.994-1.177)								
	RAPS	13	0.092	0.030	2.55E-03	1.096(1.033-1.164)								
	MR-PRESSO	13	0.092	0.030	9.61E-03	1.097(1.034-1.163)								
**CandidatusSoleaferrea**	IVW	7	-0.068	0.028	1.58E-02	0.934(0.884-0.987)	3.276	0	0.773	-0.010	0.025	0.708	4.422	0.811
	ML	7	-0.070	0.029	1.69E-02	0.933(0.881-0.988)	3.149	0	0.790					
	MR Egger	7	0.039	0.273	8.91E-01	1.040(0.610-1.774)	3.119	0	0.682					
	Weighted median	7	-0.063	0.038	9.10E-02	0.938(0.872-1.010)								
	RAPS	7	-0.015	0.025	5.52E-01	0.985(0.939-1.034)								
	MR-PRESSO	7	-0.068	0.021	1.71E-02	0.934(0.897-0.973)								
**Ruminococcaceae UCG014**	IVW	7	0.114	0.039	3.06E-03	1.121(1.039-1.210)	5.583	0	0.472	0.005	0.009	0.653	7.279	0.562
	ML	7	0.118	0.041	3.68E-03	1.126(1.039-1.219)	5.317	0	0.504					
	MR Egger	7	0.066	0.110	5.77E-01	1.068(0.861-1.325)	5.339	6.4	0.376					
	Weighted median	7	0.096	0.052	6.61E-02	1.100(0.994-1.219)								
	RAPS	7	0.089	0.033	7.43E-03	1.093(1.024-1.167)								
	MR-PRESSO	7	0.114	0.037	2.19E-02	1.121(1.042-1.206)								
**Adult-onset asthma**														
**Akkermansia**	IVW	6	-0.127	0.056	2.47E-02	0.881(0.789-0.984)	3.119	0	0.682	-0.009	0.015	0.602	4.396	0.704
	ML	6	-0.130	0.058	2.63E-02	0.878(0.783-0.985)	3.009	0	0.699					
	MR Egger	6	-0.037	0.168	8.36E-01	0.964(0.693-1.340)	2.799	0	0.592					
	Weighted median	6	-0.147	0.072	4.07E-02	0.863(0.749-0.994)								
	RAPS	6	-0.130	0.061	3.39E-02	0.878(0.779-0.990)								
	MR-PRESSO	6	-0.127	0.045	3.61E-02	0.881(0.807-0.961)								
**Childhood-onset asthma**														
**Collinsella**	IVW	4	0.340	0.124	6.04E-03	1.404(1.102-1.790)	0.130	0	0.988	-0.001	0.028	0.981	0.220	0.989
	ML	4	0.340	0.129	8.48E-03	1.405(1.091-1.810)	0.120	0	0.989					
	MR Egger	4	0.350	0.408	4.81E-01	1.419(0.638-3.157)	0.130	0	0.937					
	Weighted median	4	0.339	0.150	2.36E-02	1.404(1.047-1.883)								
	RAPS	4	0.357	0.117	2.19E-03	1.429(1.137-1.796)								
	MR-PRESSO	4	0.340	0.026	9.44E-04	1.404(1.335-1.477)								
**RuminococcaceaeUCG014**	IVW	7	0.255	0.099	1.01E-02	1.291(1.063-1.568)	7.620	21.3	0.267	0.015	0.025	0.585	10.516	0.334
	ML	7	0.270	0.094	4.17E-03	1.309(1.089-1.575)	7.196	16.6	0.303					
	MR Egger	7	0.098	0.289	7.49E-01	1.103(0.625-1.945)	7.135	29.9	0.211					
	Weighted median	7	0.192	0.121	1.12E-01	1.211(0.956-1.534)								
	RAPS	7	0.213	0.076	4.86E-03	1.237(1.067-1.435)								
	MR-PRESSO	7	0.255	0.099	4.22E-02	1.291(1.063-1.568)								
**Moderate-severe asthma**														
**FamilyXIIIAD3011group**	IVW	12	-0.378	0.111	6.93E-04	0.685(0.551-0.853)	5.846	0	0.883	-0.012	0.042	0.787	6.927	0.899
	ML	12	-0.377	0.115	1.07E-03	0.686(0.547-0.860)	5.599	0	0.899					
	MR Egger	12	-0.238	0.517	6.55E-01	0.788(0.286-2.171)	5.769	0	0.834					
	Weighted median	12	-0.277	0.147	5.95E-02	0.758(0.568-1.011)								
	RAPS	12	-0.300	0.111	6.63E-03	0.741(0.596-0.920)								
	MR-PRESSO	12	-0.378	0.081	7.01E-04	0.685(0.584-0.804)								
**Eisenbergiella**	IVW	9	0.273	0.098	5.28E-03	1.314(1.084-1.591)	10.590	24.5	0.226	-0.094	0.078	0.265	13.517	0.29
	ML	9	0.288	0.090	1.28E-03	1.334(1.120-1.590)	10.016	20.1	0.264					
	MR Egger	9	1.157	0.736	1.60E-01	3.180 (0.752-13.450)	8.753	20	0.271					
	Weighted median	9	0.209	0.121	8.44E-02	1.233(0.972-1.563)								
	RAPS	9	0.310	0.087	3.73E-04	1.363(1.149-1.617)								
	MR-PRESSO	9	0.273	0.098	2.36E-02	1.314(1.084-1.591)								
**Ruminiclostridium6**	IVW	15	-0.248	0.091	6.71E-03	0.780(0.652-0.934)	11.048	0	0.682	-0.008	0.020	0.687	12.764	0.688
	ML	15	-0.246	0.094	9.17E-03	0.782(0.650-0.941)	10.884	0	0.695					
	MR Egger	15	-0.164	0.224	4.77E-01	0.849(0.548-1.316)	10.878	0	0.621					
	Weighted median	15	-0.116	0.126	3.57E-01	0.890(0.696-1.140)								
	RAPS	15	-0.258	0.098	8.62E-03	0.773(0.637-0.937)								
	MR-PRESSO	15	-0.248	0.081	8.62E-03	0.780(0.665-0.915)								

**Note**: Estimates with 95% CI are expressed as odds ratios for asthma, and its phenotypes in genetically predicted gut microbiota. The Cochran Q-test was used to assess the heterogeneity between the SNP-specific estimates, MR-Egger regression, and MR-PRESSO to test for evidence of pleiotropy.

**Abbreviations**: MR, Mendelian Randomization; SNP, single-nucleotide polymorphism; SE, Standard error; OR, Odds ratios; CI, Confidence interval; df, Degree of freedom; SE, Standard error; RSSobs, observed residual sum of squares; IVW: Inverse variance weighting; ML: Maximum likelihood; RAPS, Robust Adjusted Profile Score; MR-PRESSO, Mendelian Randomization Pleiotropy RESidual Sum and Outlier.

### Effects of genetically predicted gut microbiota on asthma phenotypes

As for asthma phenotypes, the IVW method supported the causal associations between genetically predicted *Akkermansia* (*OR*=0.881; 95%*CI*=0.789–0.984; *P*=2.47E-02) and adult-onset asthma, and between genetically predicted *Collinsella* (*OR*=1.404; 95%*CI*=1.102–1.790; *P*=6.04E-03), *RuminococcaceaeUCG014* (*OR*=1.291; 95%*CI*=1.063–1.568; *P*=1.01E-02) and childhood-onset asthma. Similar results were also obtained in the ML, Weighted median, RAPS and MR-PRESSO methods. Furthermore, the decrease in moderate-severe asthma risk could attribute to the increase of genetically predicted *FamilyXIIIAD3011group* (*OR*=0.685; 95%*CI*=0.551–0.853; *P*=6.93E-04) and *Ruminiclostridium6* (*OR*=0.780; 95%*CI*=0.652–0.934; *P*=6.71E-03), and the increase of moderate-severe asthma risk could attribute to the increase of genetically predicted *Eisenbergiella* (OR=1.314; 95%*CI*=1.084–1.591; *P*=5.28E-03); and ML, RAPS, and MR-PRESSO methods also yielded the same results. Detailed information on the MR results can be seen in Additional file 2: **Table S5**.

### Sensitivity analyses

**Figure 1:**
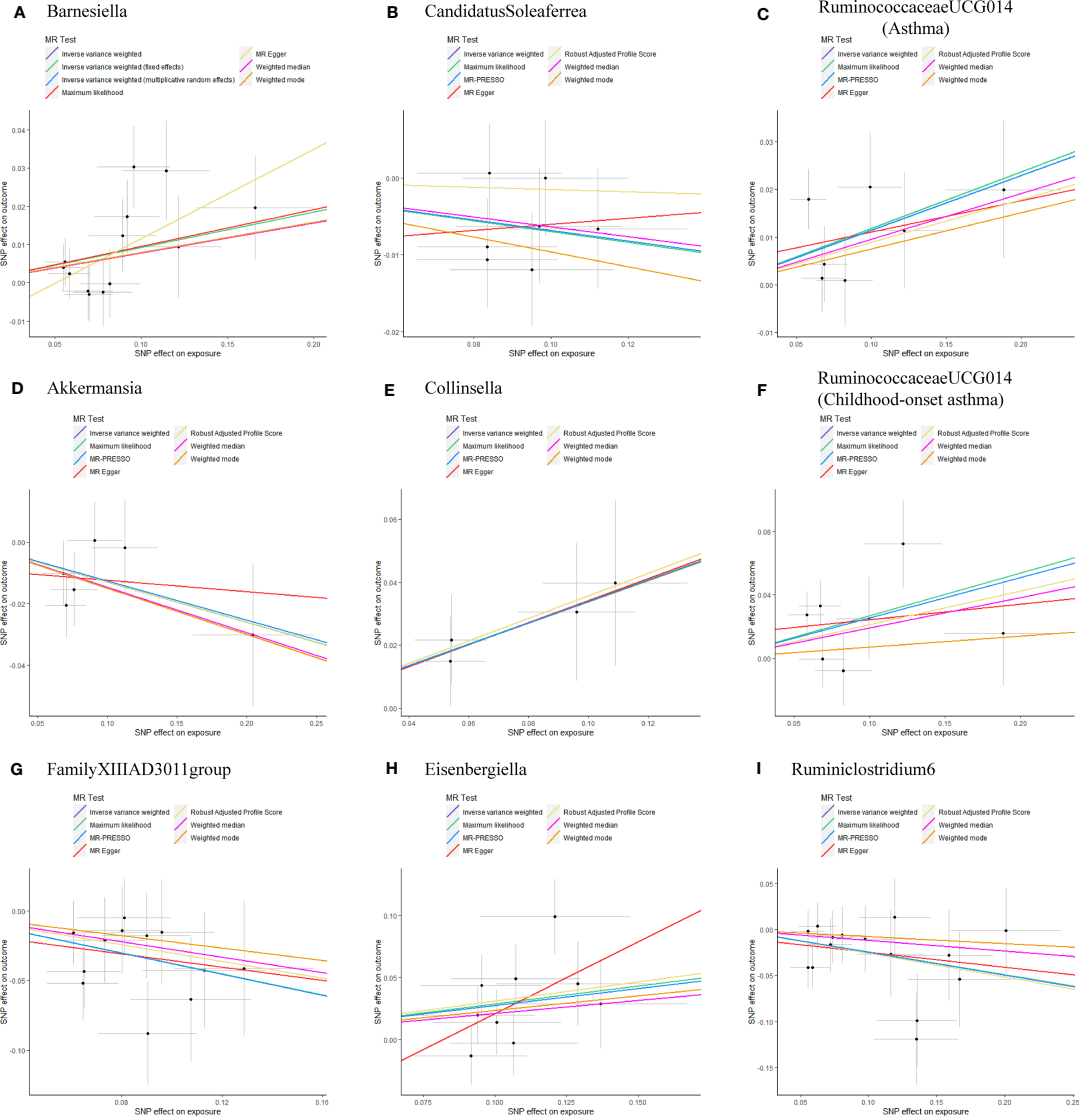
Scatterplots of potential effects of SNPs on Gut microbiota versus Asthma and its phenotypes **(A)** Barnesiella; **(B)** CandidatusSoleaferrea; **(C)** RuminococcaceaeUCG014(Asthma); **(D)** Akkermansia; **(E)** Collinsella; **(F)** RuminococcaceaeUCG014(Childhood-onset asthma); **(G)** FamilyXIIIAD3011group; **(H)** Eisenbergiella; **(I)** Ruminiclostridium6. Scatter plots presented the per-allele association with outcome risk plotted against the per-allele association with one standard deviation of exposure (with vertical and horizontal purple lines showing the 95% CI for each SNP). Analyses were conducted using the Inverse Variance Weighting (IVW), Weighted median, Wald ratio, Robust Adjusted Profile Score (RAPS), MR Egger, MR-PRESSO, and Maximum likelihood (ML) methods. The slope of each line corresponding to the estimated MR effect per method. CI, confidence interval; SNP, single-nucleotide polymorphism; MR-PRESSO, Mendelian Randomization Pleiotropy RESidual Sum and Outlier.

**Figure 2:**
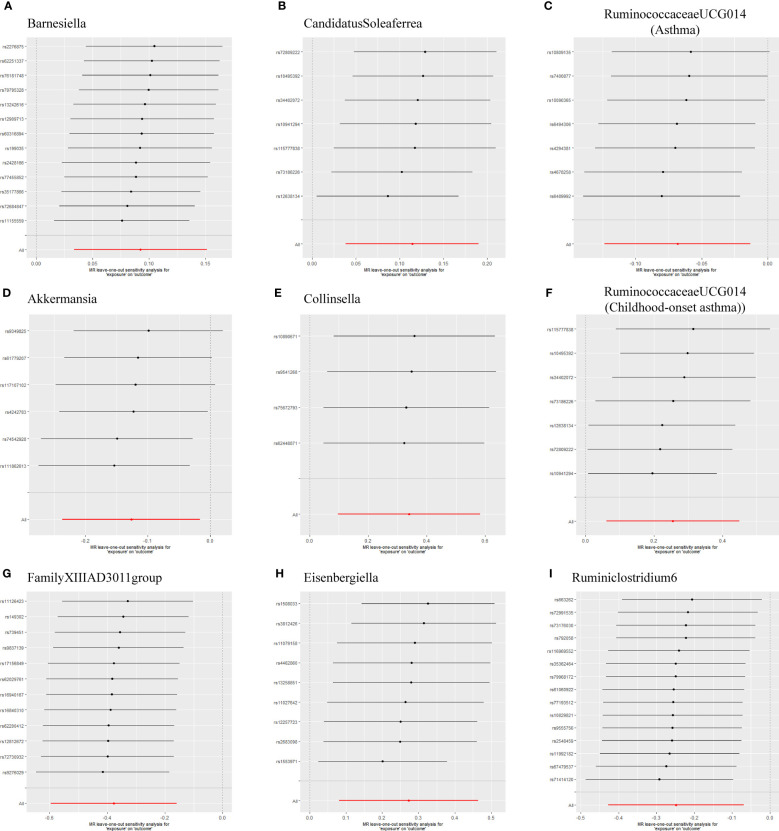
Leave-one-out sensitivity based on IVW model for Gut microbiota on Asthma and its phenotypes **(A)** Barnesiella; **(B)** CandidatusSoleaferrea; **(C)** RuminococcaceaeUCG014(Asthma); **(D)** Akkermansia; **(E)** Collinsella; **(F)** RuminococcaceaeUCG014(Childhood-onset asthma); **(G)** FamilyXIIIAD3011group; **(H)** Eisenbergiella; **(I)** Ruminiclostridium6. The IVW causal estimate and how the overall estimate (red horizontal line) was disproportionately driven, which is influenced by the removal of a single variant (black horizontal line), were visualized. There was no evidence of obvious heterogeneity, indicating that no specific SNP alone accounted for the association of gut microbiota on Periodontitis. The results suggested that there was no individual SNP with a strong influence on the overall effect. SNP, single-nucleotide polymorphism; IVW, Inverse variance weighted.

As shown in **Table 1**, the evidence of horizontal pleiotropy for SNPs was not found by using the MR-Egger regression intercept test, Cochran Q statistic and I^2^ statistic indicated low heterogeneity and more reliability of these SNPs, and MR-PRESSO global test showed that no potential outlier could affect our estimation substantially, detailed information can be seen in **Additional file 2: Table S5**. Furthermore, the funnel plots displayed general symmetry, indicating little evidence of heterogeneity (**Additional file 1: Figure S2**). Scatter plots was used to show the estimated effect sizes for SNPs of gut microbiota on asthma and its phenotypes (**Figure 1**). Beyond which, we used IVW methods to run the leave-one-out sensitivity analysis, and the results were similar after excluding individual SNPs from the study, indicating that no single SNP had an exorbitant influence on the total estimations (**Figure 2**). In the PhenoScanner search, we found that rs9276029, and rs2548459 were associated with alcohol intake, forced expiratory volume in 1-second, and peak expiratory flow (**Additional file 2: Table S6**). We re-analyzed after eliminating these SNPs, the results indicated that *FamilyXIIIAD3011group* (*OR*=0.659; 95%*CI*=0.523–0.831; *P*=4.12E-04) and *Ruminiclostridium6* (*OR*=0.771; 95%*CI*=0.642–0.927; *P*=5.70E-03) still reduced the risk of developing moderate to severe asthma, detailed information can be seen in **Additional file 2: Table S7**. The forest plots were shown in **Additional file 1: Figure S3**.

### Effects of genetically predicted asthma and its phenotypes on gut microbiota

In the reverse MR analysis, 86 genome-wide significance level (*P* < 5 × 10^–8^) and independent SNPs were proposed as IVs for asthma, and 12 SNPs for moderate-severe asthma (R^2^ < 0.001 and clump window > 10,000 kb) Bidirectional analysis for the adult-onset asthma and childhood asthma could not be conducted due to insufficient independent SNPs as IVs (0 and 1 SNP, respectively),detailed information can be seen in **Additional file 2: Table S8**.In the absence of heterogeneity and pleiotropy, the results suggest that genetically predicted asthma does not increase or decrease the risk of developing *Barnesiella*, *RuminococcaceaeUCG014*, and *CandidatusSoleaferrea*. Likewise, genetically predicted moderate-severe asthma was not associated with *FamilyXIIIAD3011group*, *Ruminiclostridium6*, and *Eisenbergiella* (**Table 2**).

**Table 2:** MR results of causal relationships between Asthma and the Gut microbiota.

*Gut microbiota*	*MR Results*						*Heterogeneity*		*Horizontal pleiotropy*		
	*Methods*	*SNPs*	*Beta*	*SE*	*P-* *value*	*OR (95% CI)*	*Cochran’s Q*	*P-* *value*	*Egger* *intercept*	*SE*	*P-* *value*
**Asthma**											
**Barnesiella**	IV	52	0.030	0.031	3.30E-01	1.030(0.9710-1.094)	38.832	0.894	0.001	0.005	0.972
	MR Egger	52	0.032	0.077	6.75E-01	1.033(0.889-1.200)	38.830	0.874			
	Weighted median	52	0.102	0.076	1.84E-01	1.107(0.955-1.284)					
**CandidatusSoleaferrea**	IVW	51	-0.004	0.054	9.39E-01	0.996(0.896-1.107)	50.081	0.510	0.002	0.005	0.722
	MR Egger	51	0.050	0.137	7.17E-01	1.051(0.803-1.376)	49.953	0.475			
	Weighted median	51	0.184	0.139	1.92E-01	1.202(0.915-1.578)					
**Ruminococcaceae UCG014**	IVW	52	-0.001	0.031	9.71E-01	0.999(0.941-1.061)	51.554	0.452	-0.005	0.005	0.328
	MR Egger	52	0.068	0.077	3.78E-01	1.071(0.921-1.244)	50.569	0.451			
	Weighted median	52	0.083	0.063	1.89E-01	1.087(0.961-1.228)					
**Moderate-severe asthma**											
**FamilyXIIIAD3011group**	IVW	12	0.007	0.023	7.53E-01	1.007(0.963-1.054)	10.823	0.458	0.021	0.023	0.380
	MR Egger	12	-0.119	0.139	4.13E-01	0.888(0.676-1.167)	9.980	0.442			
	Weighted median	12	-0.018	0.054	7.44E-01	0.982(0.883-1.092)					
**Eisenbergiella**	IVW	12	-0.002	0.040	9.56E-01	0.998(0.922-1.080)	13.412	0.267	0.031	0.040	0.457
	MR Egger	12	-0.192	0.249	4.57E-01	0.825(0.507-1.344)	12.653	0.244			
	Weighted median	12	-0.033	0.076	6.71E-01	0.968(0.834-1.122)					
**Ruminiclostridium6**	IVW	12	-0.034	0.035	3.27E-01	0.966(0.902-1.035)	9.422	0.583	-0.016	0.034	0.642
	MR Egger	12	0.066	0.211	7.63E-01	1.068(0.706-1.615)	9.192	0.514			
	Weighted median	12	0.037	0.074	6.26E-01	1.038(0.898-1.200)					

Note: Estimates with 95% CI are expressed as odds ratios for gut microbiota in genetically predicted asthma and its phenotypes. The Cochran Q-test was used to assess the heterogeneity between the SNP-specific estimates, MR-Egger regression to test for evidence of pleiotropy.

Abbreviations: MR, Mendelian Randomization; SNP, single-nucleotide polymorphism; SE, Standard error; OR, Odds ratios; CI, Confidence interval; df, Degree of freedom; SE, Standard error; RSSobs, observed residual sum of squares; IVW: Inverse variance weighting.

## Discussion

To our knowledge, this two-sample MR study, for the first time, examined the causal associations between gut microbiota and asthma by using publicly available genetic databases. Our findings revealed that several microbial genera were causally associated with asthma and its phenotypes, which enhanced the understanding regarding the role of gut microbiota in the pathology of asthma, providing new insights into the prevention and diagnosis of asthma.

The gut is the most colonized human organ with up to 100 trillion microbes, approximately 10 times the number of human cells (36), gut microbes are typically dominated by five phyla, including *Bacteroidetes*, *Firmicutes*, *Proteobacteria*, *Actinobacteria*, and *Tenericutes (* 37). They confer diverse functions to the host, including vitamin production, absorption of ions, protection against pathogens, histological development, and enhanced immune functions (38). Although the pathways through which the gut microbiota affects the lung microbiota are not fully understood, it appears that intestinal and respiratory disorders show similar clinical alterations, and an inflammatory transfer from the gut to the lung may take place (39). As a result, the disturbances of this bidirectional exchange should be connected to the increased emergence of airway illnesses such as asthma (40). Accumulating evidence highlighted the importance of the cross-talk between gut and lung, the so-called gut-lung axis, in the maintenance of immune homeostasis (41). It seemed that gut microbiota metabolites could be an important entry point in the exploration of asthma pathogenesis, short chain fatty acids, polyunsaturated fatty acids and bile acids have been shown to influence immune function by promoting the growth or maturity of certain immune cell populations (42).

The mechanisms underlying the link between gut microbiota and asthma involve complex metabolic and immune interactions of microbes, metabolites, and host immune responses (40). It was suggested that the commensal bacteria in the gut help to shape the maturation of both innate and adaptive immunity in early life, which would produce profound effects on individual’s asthma susceptibility and pathobiology. An early *in vivo* study of mouse model showed polysaccharide A from Bacteroides fragilis could induce and ligate TLR2 on plasmacytoid dendritic cells, which is priming IL-10 producing T cells with potential of anti-inflammatory properties (43). Additionally, another *in vivo* study using an HDM-challenged mouse model found that high-fiber diet increases the abundance of Bacteroides and Bifidobacterium, which can digest the fiber and produce SCFAs, such as butyrate (44). And butyrate can decrease excessive inflammation through downregulating the secretion of pro-inflammatory mediators and activating IL-10 producing T cells and macrophages (45). A very recent study also demonstrated that infants who are breastfed and given Bifidobacterium infantis EVC001 has reduced intestinal TH2 and TH17 cytokines while increased IFN-β level (44).

Previous observational and genetic studies have shown an association between adult BMI and adult-onset asthma (46). In China, recent study identified nine genome-wide significant novel loci for FEV1, six for FVC and three for FEV1/FVC in the CKB. FEV1 and FVC showed significant negative genetic correlation with obesity traits in both the CKB and UKB (47). However, one study has shown that the shared genetic component between childhood BMI and childhood-onset asthma is not driven by the genetic component of adult BMI. They identified one shared causal genomic region between BMI and asthma in childhood that mapped to gene AMN (46). To sum up, obesity is a potential confounder between gut microbiome and asthma/lung function. Our study performed statistical analysis by excluding SNPS associated with obesity (PhenoScanner database), which makes our results even more credible.

Alterations in gut microbial composition may have a notable effect on respiratory diseases, such as asthma, by shaping microbial communities and modulating the metabolic and immune response, growing evidence suggests that gut microbiota in early life is associated with childhood asthma development (18, 48, 49). Data from Canadian Healthy Infant Longitudinal Development Study (CHILD) demonstrated the relative abundance of the genera *Lachnospira*, *Faecalibacterium*, *Veillonella* (Phylum *Firmicutes*) and *Rothia* (Phylum *Actinobacteria*) was considerably lowered in the gut microbiome of infants at risk for asthma in the first 100 days of life (48). Another cohort study found that the opposing shift in the relative abundance of *Lachnospira* and *Clostridium* (Phylum *Firmicutes*) in the early life stage was associated with asthma development at preschool age (18). At 3 months the abundance of the genera, *Lachnospira* was decreased, whereas *Clostridium* was increased in asthmatics, and the *Lachnospira*/*Clostridium* ratio may serve as a potential early life biomarker to predict asthma development (18). A recent metabolomics-based study made a comparative analysis of stool samples from asthma children and healthy children both aged 4 to 7 years, indicating that children with allergic airway illnesses tended to have a much lower abundance of the *Firmicutes (* 50). The results suggested that childhood rhinitis and asthma may be caused by a decrease in certain gut microbes in *Firmicutes*, which are involved in the up-regulation of fecal amino acids (50). All above studies suggest that taxa belonging to the phyla *Firmicutes* or *Actinobacteria* should play an important role in the pathogenesis of asthma. Particularly, our present study is the first to provide genetic causal evidence to confirm and enrich the current knowledge. We found that *Candidatus, Soleaferrea* and *RuminococcaceaeUCG014* (Phylum *Firmicutes*, Class *Clostridia*, order *Clostridiales*) were correlated with the risk of asthma, among which, *Collinsella* (Phylum *Actinobacteria*) and *RuminococcaceaeUCG014* increased the risk of childhood-onset asthma, and *Eisenbergiella* and *Ruminiclostridium6* (Phylum *Firmicutes*, class *Clostridia*) were associated with the risk of moderate-severe asthma. In South of China, a cross-sectional study investigating the gut microbiome profile in adults found that *Ruminococcus gnavus* were enriched in newly diagnosed asthmatic patients compared to healthy controls (51). In a twin cohort study, researchers discovered a correlation between the abundance of fecal *Ruminococcus gnavus* and the development of allergies, particularly respiratory allergies (52). Likewise, *RuminococcaceaeUCG014* has a positive effect against asthma as well as childhood-onset asthma in the present study. There are fewer studies on *Collinsella* and allergic disease, a recent case-control study observed significant increases in the numbers of *Collinsella*, *Ruminococcus*, and *Akkermansia* in the food allergy group compared to the control group, at the genus level (53), and another epidemiological study found that indoor *Collinsella* was positively associated with asthma, rhinitis and eczema among preschool children (54). All these findings are quite similar to ours. A study from the US birth cohort found that the neonates with the lowest relative abundance of *Akkermansia* have the highest risk of developing asthma at age of four years, and we got the consistent findings among adults in the present study (55). *Eisenbergiella* was found to be associated with nasal symptoms in patients with allergy rhinitis (56), our result demonstrated that *Eisenbergiella* was correlated with the risk of moderate-severe asthma. Furthermore, we found genetic causality between *Barnesiella*, *CandidatusSoleaferrea* and asthma, and between *FamilyXIIIAD3011group*, *Ruminiclostridium6* and moderate-severe asthma. Research on these genera is still relatively scarce, and more research is needed on their role in allergic diseases in the future.

This MR study has the following advantages. The use of the MR technique reduced the interference of confounding factors and false causality in the results, which is the first strength. To the best of our knowledge, this is the first MR analysis investigating the causal association between gut microbiota and asthma. Our results offer a theoretical foundation for the subsequent investigation into the regulation mechanism of a single strain on asthma. Second, the current analysis makes full use of the comprehensive GWAS data that are publicly available, which promised a high sample sizes; making our study have necessary power to estimate reliable and lifelong causality. Third, because we conducted MR analysis on gut microbiota at the genus level, it should be possible to pinpoint specific bacterial strains that are indeed causally related to asthma. Our study does have some limitations, though. First, our findings were not robust to Bonferroni-adjusted significance, but MR analysis serves as a hypothesis-driven study testing epidemiologically established associations based on enough physiological evidence. Second, the number of genetic loci found in gut microbiota GWAS is still small, our IVs are suspected of being weak tool variables, which may reduce the statistical power of our MR study. Third, a tiny percentage of the microbiota data were of different races, despite the fact that the majority of the data included in our analysis were European, which may to some extent throw off our estimates.

## Conclusion

In summary, by performing two sample MR analyses, our study is the first to provide comprehensive screening data regarding the causal associations of gut microbes with asthma as well as its three phenotypes; by contrast, the reverse MR analysis didn’t establish reverse causal link from asthma and its three phenotypes to the gut microbiota genus. The findings underscore the critical role of gut microbiota in asthma’s pathogenesis, which would be of significance in clinical application; the gut microbes identified may be the potential therapeutic targets for asthma preventing and clinical treating.

## Data availability statement

All data used in the present study were obtained from genome-wide association study summary statistics which were publicly released by genetic consortia. Gut microbiota: https://www.ebi.ac.uk/gwas/publications/33462485. Asthma, adult-onset asthma and childhood-onset asthma: https://www.ebi.ac.uk/gwas/publications/31619474. Moderate-severe asthma: https://www.ebi.ac.uk/gwas/publications/30552067. All datasets generated for this study are included in the article/Additional files.

## Author contributions

RL, QG, and SL designed the study, contributed to the data analysis, and wrote the manuscript. RL, QG, JZ, WK, RYL, LH, ZL, YC, and AZ contributed to the data analysis and data interpretation. JW, YY, and SL contributed to manuscript writing and revision of the manuscript. All authors contributed to the article and approved the submitted version.
