# Supplementary material for: Assessing causal relationships between gut microbiota and asthma: evidence from two sample Mendelian randomization analysis
Source: Front Immunol. 2023 Jul 19;14:1148684. doi: 10.3389/fimmu.2023.1148684 (PMC10394653; doi:10.3389/fimmu.2023.1148684)
Supplement: Supplementary file 1 [file Table_1.docx]

**Assessing causal relationships between Gut microbiota and Asthma: evidence from a two sample Mendelian randomization analysis**

Rong Li^a#^, Qi Guo^b#^, Jian Zhao^a^, Wenhui Kang^a^, Ruoyu Lu^a^, Zichong Long^a^, Lili Huang^a^, Yiting Chen^a^, Anda Zhao^c^, Jinhong Wu^d^, Yong Yin^d^*****, Shenghui Li^a^*****

^a^ MOE-Shanghai Key Laboratory of Children’s Environmental Health, School of Public Health, Shanghai Jiao Tong University School of Medicine, Shanghai 200025, China

^b^ Minhang District Center for Disease Control and Prevention, Shanghai 201101, China

^c^ Shanghai Ninth People's Hospital, Shanghai Jiao Tong University School of Medicine, Shanghai 200011, China

^d^ Shanghai Children's Medical Center, Shanghai Jiao Tong University School of Medicine, Shanghai 200127, China

**^#^ Rong Li and Qi Guo contributed equally to this work.**

**^*^ Correspondence:**

Shenghui Li, MD, PhD, School of Public Health, Shanghai Jiao Tong University, 227 South Chongqing Road, Huangpu District, Shanghai, 200025, China. Tel: +86-21-63846590; E-mail: [lsh9907@163.com](mailto:lsh9907@163.com), [submission9907@163.com](mailto:submission9907@163.com)

Yong Yin, MD, PhD, Department of Respiratory Medicine, Shanghai Children's Medical Center, Shanghai Jiao Tong University School of Medicine, 1678 Dongfang Road, Pudong New Area, Shanghai, 200127, China. Tel: +86-21-38626161; E-mail: yinyong9999@163.com

**Additional file 1**

**Additional file 1: Table S1 Study design to investigate the association between gut microbiota and asthma basing on two sample Mendelian randomization assumptions**

**Additional file 1: Table S2 Details of the GWAS data for Gut microbiota, Asthma, and its phenotypes**

**Additional file 1: Figure S1 Funnel plots for MR analyses of the causal effect of Gut microbiota on Asthma, and its phenotypes**

**Additional file 1: Figure S2 Funnel plots for MR analyses of the causal effect of Gut microbiota on Asthma, and its phenotypes**

**Additional file 1: Figure S3 Forest plot for the association between Gut microbiota on Asthma, and its phenotypes**

| **Additional file 1: Table S1 Detailed information on the classification of Gut microbiota** | |
| --- | --- |
| ***Classification*** | ***Gut microbiota and its code*** |
| **Genus** | Clostridiuminnocuumgroup.id.14397、Eubacteriumbrachygroup.id.11296、Eubacteriumcoprostanoligenesgroup.id.11375、Eubacteriumeligensgroup.id.14372、Eubacteriumfissicatenagroup.id.14373、Eubacteriumhalliigroup.id.11338、Eubacteriumnodatumgroup.id.11297、Eubacteriumoxidoreducensgroup.id.11339、Eubacteriumrectalegroup.id.14374、Eubacteriumruminantiumgroup.id.11340、Eubacteriumventriosumgroup.id.11341、Eubacteriumxylanophilumgroup.id.14375、Ruminococcusgauvreauiigroup.id.11342、Ruminococcusgnavusgroup.id.14376、Ruminococcustorquesgroup.id.14377、Actinomyces.id.423、Adlercreutzia.id.812、Akkermansia.id.4037、Alistipes.id.968、Allisonella.id.2174、Alloprevotella.id.961、Anaerofilum.id.2053、Anaerostipes.id.1991、Anaerotruncus.id.2054、Bacteroides.id.918、Barnesiella.id.944、Bifidobacterium.id.436、Bilophila.id.3170、Blautia.id.1992、Butyricicoccus.id.2055、Butyricimonas.id.945、Butyrivibrio.id.1993、CandidatusSoleaferrea.id.11350、Catenibacterium.id.2153、ChristensenellaceaeR.7group.id.11283、Clostridiumsensustricto1.id.1873、Collinsella.id.815、Coprobacter.id.949、Coprococcus1.id.11301、Coprococcus2.id.11302、Coprococcus3.id.11303、DefluviitaleaceaeUCG011.id.11287、Desulfovibrio.id.3173、Dialister.id.2183、Dorea.id.1997、Eggerthella.id.819、Eisenbergiella.id.11304、Enterorhabdus.id.820、Erysipelatoclostridium.id.11381、ErysipelotrichaceaeUCG003.id.11384、Escherichia.Shigella.id.3504、Faecalibacterium.id.2057、FamilyXIIIAD3011group.id.11293、FamilyXIIIUCG001.id.11294、Flavonifractor.id.2059、Fusicatenibacter.id.11305、Gordonibacter.id.821、Haemophilus.id.3698、Holdemanella.id.11393、Holdemania.id.2157、Howardella.id.2000、Hungatella.id.11306、Intestinibacter.id.11345、Intestinimonas.id.2062、Lachnoclostridium.id.11308、Lachnospira.id.2004、LachnospiraceaeFCS020group.id.11314、LachnospiraceaeNC2004group.id.11316、LachnospiraceaeND3007group.id.11317、LachnospiraceaeNK4A136group.id.11319、LachnospiraceaeUCG001.id.11321、LachnospiraceaeUCG004.id.11324、LachnospiraceaeUCG008.id.11328、LachnospiraceaeUCG010.id.11330、Lactobacillus.id.1837、Lactococcus.id.1851、Marvinbryantia.id.2005、Methanobrevibacter.id.123、Odoribacter.id.952、Olsenella.id.822、Oscillibacter.id.2063、Oscillospira.id.2064、Oxalobacter.id.2978、Parabacteroides.id.954、Paraprevotella.id.962、Parasutterella.id.2892、Peptococcus.id.2037、Phascolarctobacterium.id.2168、Prevotella7.id.11182、Prevotella9.id.11183、RikenellaceaeRC9gutgroup.id.11191、Romboutsia.id.11347、Roseburia.id.2012、Ruminiclostridium5.id.11355、Ruminiclostridium6.id.11356、Ruminiclostridium9.id.11357、RuminococcaceaeNK4A214group.id.11358、RuminococcaceaeUCG002.id.11360、RuminococcaceaeUCG003.id.11361、RuminococcaceaeUCG004.id.11362、RuminococcaceaeUCG005.id.11363、RuminococcaceaeUCG009.id.11366、RuminococcaceaeUCG010.id.11367、RuminococcaceaeUCG011.id.11368、RuminococcaceaeUCG013.id.11370、RuminococcaceaeUCG014.id.11371、Ruminococcus1.id.11373、Ruminococcus2.id.11374、Sellimonas.id.14369、Senegalimassilia.id.11160、Slackia.id.825、Streptococcus.id.1853、Subdoligranulum.id.2070、Sutterella.id.2896、Terrisporobacter.id.11348、Turicibacter.id.2162、Tyzzerella3.id.11335、unknownid.826、unknownid.959、unknownid.1868、unknownid.2001、unknownid.2041、unknownid.2071、unknownid.2755、unknownid.1000000073、unknownid.1000001215、unknownid.1000005472、unknownid.1000005479、unknownid.1000006162、Veillonella.id.2198、Victivallis.id.2256 |

| **Additional file 1: Table S2 Details of the GWAS data for Gut microbiota, Asthma, and its Phenotypes** | | | | | | |
| --- | --- | --- | --- | --- | --- | --- |
| ***Traits*** | ***PMID*** | ***Sample size*** | ***Cases*** | ***Controls*** | ***Consortium or study*** | ***Ancestor*** |
| Gut microbiota | 33462485 | 18,340 | — | — | BSPSPC, CARDIAb, CARDIAw, COPSAC, DanFunD16, FGFP, FOCUS, GEM_HCE_v12, GEM_HCE_v24, GEM_ICHIP_HCE, GenR, HCHS/SOL, KSCS, LLD, METSIM, MIBS, NGRC, NTR, PNP, POPCOL, RS3, SHIP, SHIP-TREND, TwinsUK | European (78%), Multiethnic (7.24%), Hispanic (5.98%), East Asian (4.42%), Middle-East (2.62%), Admixed (1.11%), AfroAmerican (0.62%) |
| Asthma | 31619474 | 394,283 | 46,802 | 347,481 | UK Biobank | European (100%) |
| Adult-onset asthma | 31619474 | 369,777 | 22,296 | 347,481 | UK Biobank | European (100%) |
| Childhood-onset asthma | 31619474 | 357,157 | 9,676 | 347,481 | UK Biobank | European (100%) |
| Moderate–severe asthma | 30552067 | 30,810 | 5,135 | 25,675 | GASP, U-BIOPRED, UK Biobank | European (100%) |

**
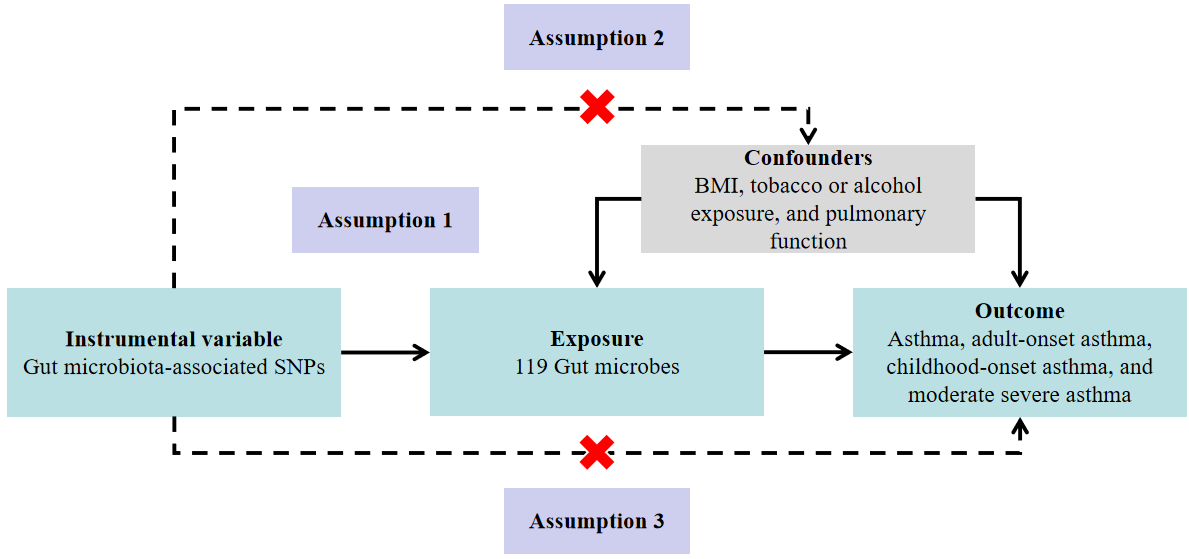
**

**Figure S1 Study design to investigate the association between gut microbiota and asthma basing on two sample Mendelian randomization assumptions.**

***Note:*** The MR study must satisfy three assumptions. Assumption 1: genetic variation used for instrumental variable is associated with exposure; Assumption 2: genetic variation is independent of confounding factors; Assumption 3: genetic variation affects the outcome only through the exposure and not through other pathways.

***Abbreviations*:** MR, Mendelian randomization; SNP, single-nucleotide polymorphism.

**
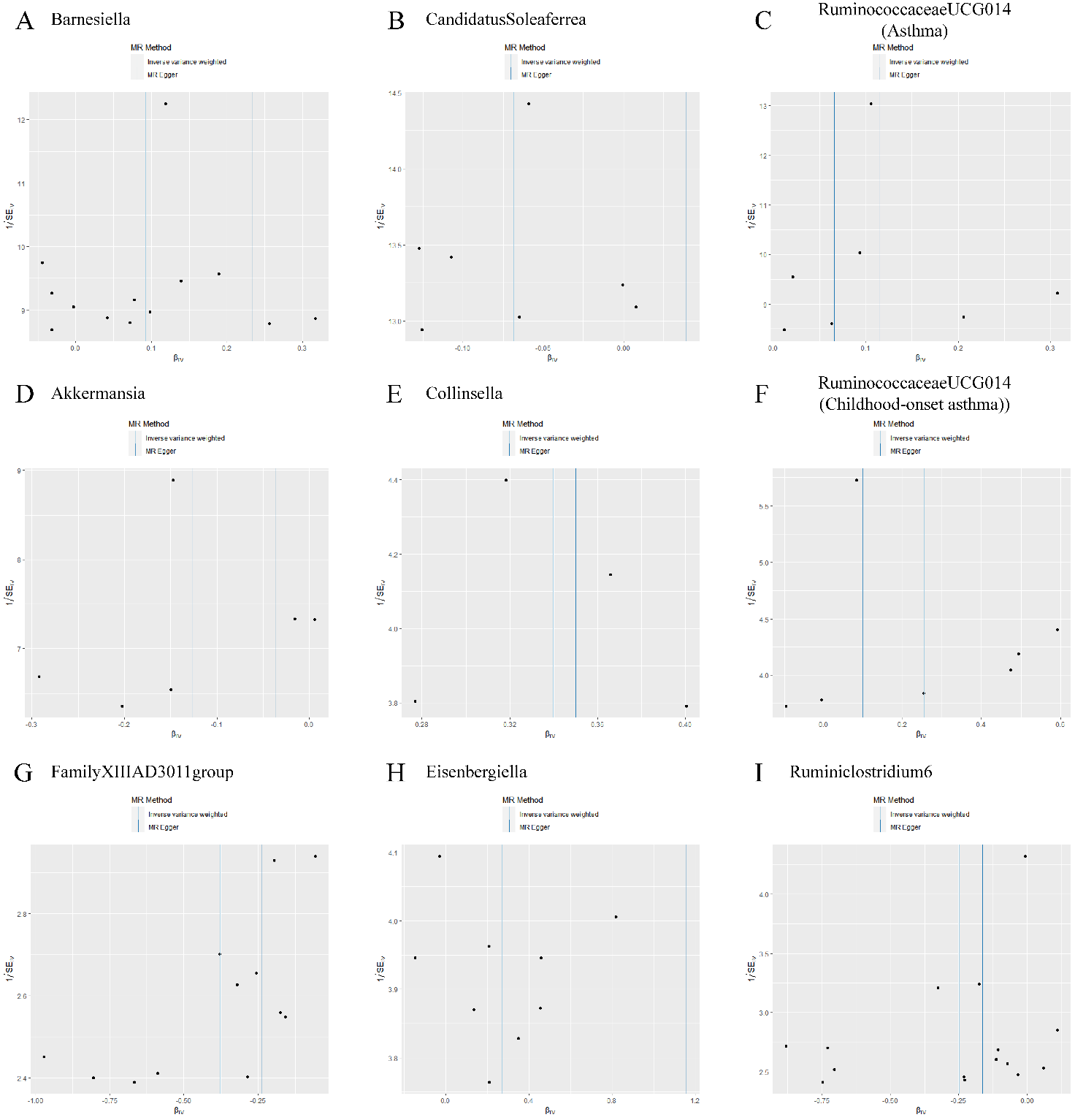
**

**Additional file 1: Figure S2** **Funnel plots for MR analyses of the causal effect of Gut microbiota on Asthma, and its phenotypes**

***Note:*** A Barnesiella; B CandidatusSoleaferrea; C RuminococcaceaeUCG014(Asthma); D Akkermansia; E Collinsella; F RuminococcaceaeUCG014(Childhood-onset asthma); G FamilyXIIIAD3011group; H Eisenbergiella; I Ruminiclostridium6. Inverse Variance Weighting (IVW) and MR Egger methods were used to detect the heterogeneity of SNP. The funnel plots showed general symmetry, suggesting little evidence of heterogeneity.

***Abbreviations:*** SNP, single-nucleotide polymorphism; MR, Mendelian Randomization.


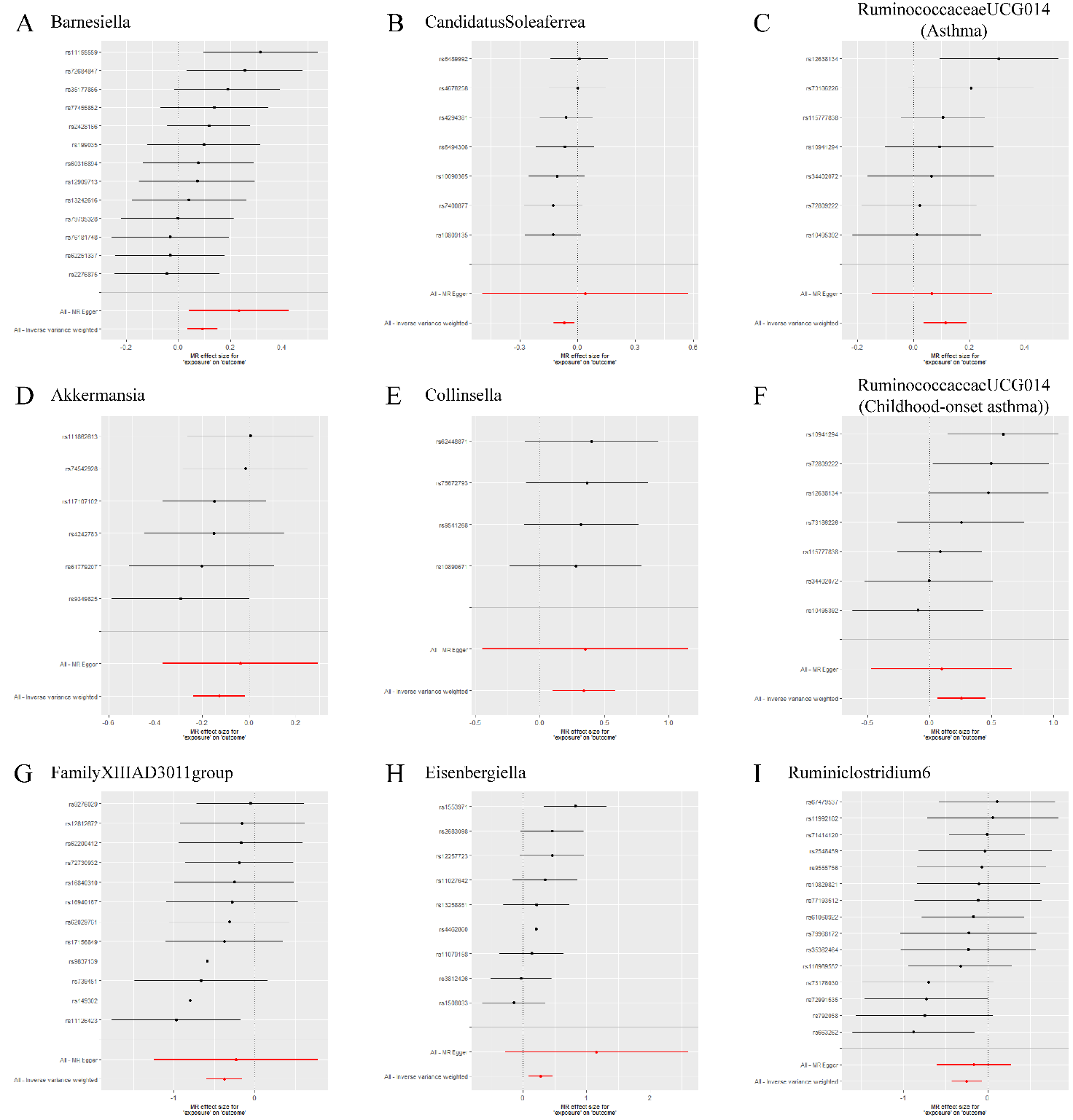


**Additional file 1: Figure S3 Forest plot for the association between Gut microbiota on Asthma, and its phenotypes**

***Note*:** A Barnesiella; B CandidatusSoleaferrea; C RuminococcaceaeUCG014(Asthma); D Akkermansia; E Collinsella; F RuminococcaceaeUCG014(Childhood-onset asthma); G FamilyXIIIAD3011group; H Eisenbergiella; I Ruminiclostridium6
